# Epidemiological Study on the Status of Nutrition-Support Therapies by Emergency Physicians in China

**DOI:** 10.1155/2019/7657436

**Published:** 2019-12-01

**Authors:** Chi Niu, Wei Guo, Wei Gu

**Affiliations:** ^1^Emergency Department, Beijing Tian Tan Hospital, Capital Medical University, Beijing, China; ^2^Emergency and Critical Care Center, ChuiYang Liu Hospital Affiliated to Tsinghua University, Beijing, China

## Abstract

**Objectives:**

This study aimed to investigate the current status of nutrition-support therapies by emergency physicians in China and to provide an evidence-based case to improve the regulation of enteral and parenteral nutrition-support therapies.

**Methods:**

Physicians from the Emergency Branch of the China Geriatrics Society were enrolled in the present survey. A questionnaire related to nutrition-support therapy, including the time, location, ways, indications, complications, and nutrition-support training for physicians was answered.

**Results:**

527 questionnaires were collected from over 300 hospitals in 25 provinces of China. The time to initiation of emergency nutrition support was often delayed. Furthermore, the treatment intensity and standardized training of physicians are weaknesses concerning nutrition support. Treatment standardization has been significantly improved, including blood glucose monitoring, precaution and management of complications, and the use of immunomodulators.

**Conclusions:**

Emergency physicians should pay attention to early identifying and providing nutrition support to those patients who need it. Finally, standardized training should be developed for emergency nutrition-support therapy.

## 1. Introduction

The incidence of malnutrition in hospitalized patients is 40–50%. Malnutrition may occur due to multiple reasons, including impaired digestive function, stress-induced accelerated metabolism, malabsorption, and nutrient loss for other factors. Chronic malnutrition can lead to a weakened immune system, severer infection symptoms, and even multiple organ failure. However, emergency physicians are often unaware of the need for early nutrition support [1], which could contribute to the subsequent treatment of patients in the early stage of many critical illnesses. Without nutritional support, patients' immune function may continue to decline, which increases the risk of secondary infection and poor prognosis.

Patients in emergency departments usually have disease characteristics that are distinct from those of generally admitted patients, such as acute onset, rapid development, and critical illness. After the acute onset of the disease, the body of the patient is in a state of stress and high metabolic decomposition, which increases nutrient and calorie consumption. Therefore, if the physician did not make an accurate assessment on the severity of the patient's disease condition and current nutrition status, malnutrition may become worse and aggravate the ongoing metabolic disorder [2, 3]. Currently, there are no specific investigations into the status of nutrition-support therapies by emergency physicians in China. Thus, the Nutrition Group of the China Geriatrics Society's Emergency Branch conducted a survey on nutrition-support therapies by emergency physicians. The present study aims to investigate the current status of nutrition support and to provide an evidence-based case to improve the regulation of enteral and parenteral nutrition-support therapies by emergency physicians in China.

## 2. Materials and Methods

Physicians from the Emergency Branch of the China Geriatrics Society were invited for the survey and further enrolled in the present survey. The survey relied on data from the Golden Data questionnaire survey platform. The questionnaire was discussed and formulated by experts of the nutrition professional committee, including over 20 items associated with nutrition-support therapy, such as the time, location, ways, indications, complications, and nutrition-support training for physicians. Questionnaires were sent to the participating hospitals by means of WET and filled out by full-time emergency physicians in each hospital.

Statistical analysis was conducted using the SPSS (version 20.0) software. All data are expressed as mean ± standard deviation, median (intertertile range), or percentage, where appropriate.

## 3. Results

During the study, more than 527 questionnaires were collected from over 300 hospitals in 25 provinces of China. 97.2% of the physicians were employed in emergency departments of level-two hospitals or higher (80.1% from level-two hospitals and 17.1% from level-three hospitals). The proportion of respondents who were middle-level or senior physicians is 83.4% (Table 1).

50% of patients receiving nutrition support were over 75 years old. Initiation of nutrition support mainly occurred in intensive care units (ICUs) and rescue rooms (44.1% and 27.5%, respectively). The proportion of nutrition support in observation rooms of the emergency department was relatively low (23.2%). Nutrition support applied even less frequently in the preliminary diagnosis area of emergency departments (5.2%) is shown in Table 1. For most patients, nutrition support was initiated when admittance to the hospital, but not immediately: 48.4% nutrition support began at above 12 hours after being admitted and 24.4% began after 24 hours of being admitted.

**Figure 1:**
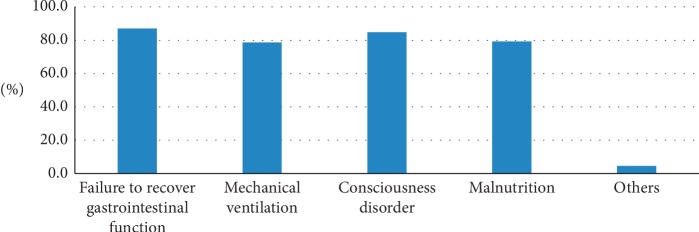
Diseases of emergency patients receiving nutrition support.

The main pathways of nutrition support were the veins (88%) and nasogastric tubes (91%) (Table 1). Emergency physicians were more willing to accept enteral nutrition; 40% of physicians reported that they rarely applied for intravenous nutrition support. Most reported diseases receiving nutrition support were failure to recover gastrointestinal function (87%), mechanical ventilation (78.6%), consciousness disorder (84.8%), and malnutrition (79.2%) (Figure 1).

**Figure 2:**
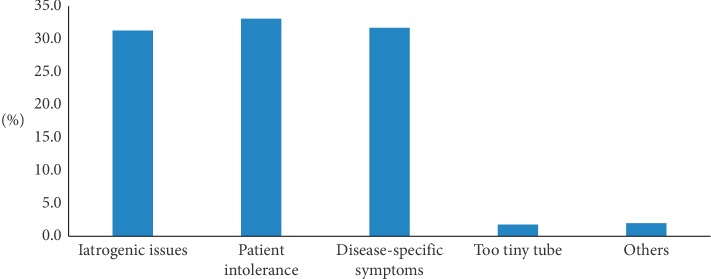
Primary reasons for inadequate feeding during nutrition-support therapy.

**Figure 3:**
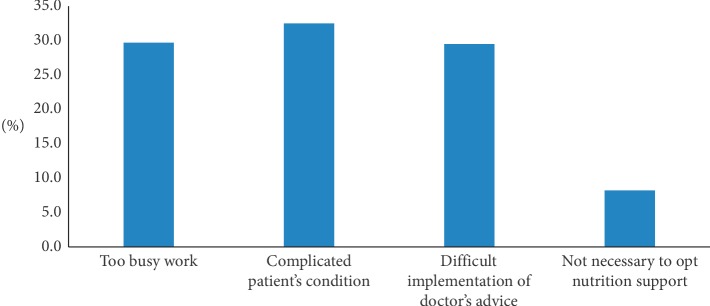
Reasons why emergency physicians were reluctant to carry out early nutrition support.

22.6% of patients had blood sugar higher than 8.3 mmol/L during nutrition-support therapy. Most physicians (90.8%) actively monitored blood glucose during treatment and controlled it with insulin. About 60% of patients experienced inadequate feeding with daily caloric intake <25 kcal/kg/d. The reasons for inadequate feeding included iatrogenic issues (31.3%), patient intolerance (33.1%), and disease-specific symptoms (31.7%) (Figure 2). The reasons that emergency physicians gave for being unwilling to administer nutrition support included feeling too busy with workload (29.7%), the patient's condition was too complicated (32.5%), there was difficulty implementing the doctor's advice (29.5%), and concluding that nutrition support was not necessary (32.5%) are shown in Figure 3.

45.7% of doctors had not received any formal training on nutrition support in the past year. However, nearly 50% doctors showed interest in participating in such training in the near future. Table 1 shows the main options for nutrition-support training including lectures (75.6%), textbooks, and reference books (72.1%) and learning from experienced doctors during ward rounds (57.7%). The proportions of emergency physicians who reported learning nutrition support from medical books and medical app software were 27.7% (books) and 17.6% (apps), respectively.

## 4. Discussion

Although the current status of emergency nutrition-support therapy in China is still inadequate, our results show that with the rapid development of emergency medicine and rapid improvement in overall treatment levels in China, nutrition support has also become more standardized. However, the time of starting nutrition support in the emergency department was often delayed. Furthermore, the treatment intensity and standardized training of physicians are weaknesses concerning nutrition support.

Elderly patients have a high incidence of acute attack during chronic disease, and the number of old-age patients in the emergency department is increasing with the intense population aging in China. Our survey shows that approximately 50% of patients who receive nutrition support were over 75 years old. These patients often suffered from multiple chronic diseases such as diabetes, hypertension, and coronary heart disease before admitted to the hospitals, at which point their nutritional status is often unsatisfactory [4]. The prevalence of malnutrition in elderly patients in emergency departments was much higher than that in the solitary older adults in the community [5]. Patients are under stress and have higher metabolic decomposition after the sudden onset of diseases, especially in the elderly population, and their consumption of nutrient and calorie will increase, which raises the risk for malnutrition. Emergency physicians should focus on nutrition support of elderly patients.

This survey shows that emergency physicians mainly initiated nutrition-support therapies in the rescue room and the emergency intensive care unit (EICU), while nutrition support was rarely initiated in the observation room. Patients often experienced delays in being transferred to the appropriate ward in China, resulting in a prolonged stay in the observation room [6]. The observation room is a special setting in emergency departments that usually treats relatively stable patients who require short-term hospitalization [7]. Moreover, the medical staff of the observation room are typically very busy, and they cannot evaluate the nutrition status of each patient nor administer nutrition support for them although studies have demonstrated that earlier enteral or parenteral nutrition support for critical patients fosters a better prognosis [8, 9]. Therefore, our results indicated that emergency physicians should pay more attention to nutrition support for observation-room patients with chronic or frequently occurring conditions.

Additionally, we found that time of initiating nutrition support for patients in emergency departments was generally delayed. Most patients were given nutrition-support therapy at 12 hours after hospital admission, and 24.4% patients were not given nutrition support until 24 hours after admission. Early nutrition support is essential to the improvement of prognosis, especially in critical and emergency patients. Early initiation of gastrointestinal nutrition can stimulate gastrointestinal secretion, maintain splanchnic blood flow stability and gastrointestinal mucosal integrity, and enhance the defense of the gastric mucosa [10, 11]. Additionally, early enteral nutrition fosters the growth of normal intestinal flora, helps intestinal bacteria secrete immunoglobulins, protects the intestinal immune and barrier functions, and prevents complications, such as bacterial translocation and gastrointestinal bleeding [12, 13]. We found that influence factors for delayed initiation of nutrition support in the emergency department were mainly related to the high workload of the emergency medical staff, complex disease conditions, and difficulty implementing the doctor's advice. We consider these as the main problems to address for improving nutrition therapy in the future.

It is necessary to develop a reasonable nutrition-support regimen for emergency physicians based on the severity of patient's condition and the functional status of the gastrointestinal tract, while the patient is still in an early disease stage [14]. Patients who cannot eat on their own should be given nasal feeding as the preferred method of enteral nutrition [15, 16]. For patients who cannot tolerate enteral nutrition or who present with enteral nutrition contraindications, such as gastrointestinal dysfunction, stress, or peptic ulcer bleeding, parenteral nutrition can be applied [17, 18]. Our results show that emergency physicians were fairly willing to opt enteral nutrition; meanwhile, 40% of physicians reported rarely administering intravenous nutrition support. 95% of doctors took interventions when adverse reactions to feeding occurred, which reflects an improvement of these emergency physicians on administering nutrition support. For the implementation of enteral and parenteral nutrition longer than one week, it is necessary to regularly assess nutritional status and disease changes and to adjust any components of the nutrition-support protocol on time to reduce the incidence of complications [19–21].

Finally, we found that 45.7% of emergency physicians had not received regular training on nutrition support in the past year. However, 50% of doctors expressed interest in participating in such training in the near future. Studies have shown that systematic nutrition-support training can improve physicians' nutrition knowledge and increase patients' access to effective nutritional treatment, which significantly improves prognosis [22, 23]. Therefore, we suggested to promote nutrition-support training for emergency doctors at all levels through various platforms and increase their ability to apply nutrition-support therapy.

With the wide application of nutrition support in clinical practice, the construction of nutrition-support team (NST) also plays a vital role. In particular, with the participation of clinical pharmacists, they can give full play to their professional skills and establish an NST together with emergency medical staff to provide patients with whole-course pharmaceutical care in clinical nutrition treatment. Clinical pharmacists can provide personalized nutrition-support programs according to different patients, especially the prescription of total nutrition mixture provided by clinical pharmacists, which not only reduces the unreasonable formula, but also reduces the burden of energy conversion for clinicians. However, it was found in our investigation that almost no NST participated in the nutrition-support treatment of emergency patients, and it is one of the main development directions of nutrition-support treatment in the future.

## 5. Conclusion

Malnutrition in emergency patients might trigger aggravation of primary diseases and increase complications; it also prolongs the average hospital stay and increases medical expenses. Therefore, emergency physicians should pay attention to early nutrition risk of patients and provide nutrition support to those who need it, which will benefit the patient's recovery and reduce complications. In future, emergency nutrition support should be focused on elderly patients with emergency malnutrition particularly. Finally, developing standardized training and an emergency nutrition-support team is needed for emergency nutrition-support therapy.

## Figures and Tables

**Table 1:** The main results of the survey.

Survey content (percentage, %)	
The doctor's level	
Senior title	52.3
Intermediate title	31.1
Primary title	16.6

Initiation of nutrition support	
EICU	44.1
Rescue rooms	27.5
Observation rooms	23.2
Diagnosis area	5.2

Pathways of nutrition support	
Parenteral nutrition	88
Enteral nutrition	91

Options for nutrition-support training	
Meeting lectures	75.6
Reference books	72.1
Experienced doctors	57.7

## Data Availability

The data used to support the findings of this study are included within the article.
